# Amniotic membranes in ophthalmology: long term data on transplantation outcomes

**DOI:** 10.1007/s10561-015-9520-y

**Published:** 2015-07-11

**Authors:** Adolfo Paolin, Elisa Cogliati, Diletta Trojan, Carlo Griffoni, Andrea Grassetto, Hossein Mostafa Elbadawy, Diego Ponzin

**Affiliations:** Treviso Tissue Bank Foundation, Piazzale Ospedale 1, 31100 Treviso, Italy; Veneto Eye Bank Foundation, Via Paccagnella 11, 30174 Venice, Italy

**Keywords:** Amniotic membrane, Collection, Preservation, Distribution, Follow-up, Long term

## Abstract

The use of amniotic membrane (AM) is a widespread clinical practice for eye surgeries and the treatment of an increasing number of ocular surface pathologies. Here we describe the AM collection methods and donor selection criteria adopted by our tissue bank to distribute 5349 amniotic membrane patches over the last 12 years for the treatment of several ocular pathologies. Specific quality control measures are described and the long term results attained using the reported procedure are presented. A case of AM utilized to treat severe ocular ulceration is also described as an example of AM transplantation. Collective data for the total amniotic membrane patches deployed to treat various ocular diseases are discussed and success rates for AM transplantations are reported. An extensive follow-up is illustrated. The results suggest that the procedures and protocols used by the Treviso Tissue Bank Foundation and Veneto Eye Bank Foundation for collection, preservation, distribution and follow-up are of an optimal standard. Accordingly, the authors conclude that the safety and efficiency of the proposed procedure for the therapeutic use of AM to treat various ocular pathologies are reproducible, with additional evidence favoring the use of AM as an alternative to conventional medical treatment for certain ocular conditions.

## Introduction

The amniotic membrane (AM) is the inner layer of the fetal membranes and is comprised of three distinct layers; epithelium, basement membrane and stroma, the innermost layer that further consists of an inner compact layer, middle fibroblast layer and an outermost spongy layer. The use of AM in patients was reported for the treatment of cicatricial pemphigoid and Stevens–Johnson syndrome (Tsubota et al. 1996), pterygium (Prabhasawat et al. 1997), persistent epithelial defects with ulceration (Lee and Tseng 1997), conjunctival surface reconstruction (Tseng et al. 1997) and ocular surface reconstruction in patients with chemical and thermal burns (Shimazaki et al. 1997). The benefits of AM can be attributed to its anti-inflammatory and anti-microbial properties (Solomon et al. 2001) and low immunogenicity (Hori et al. 2006). The earliest evidence of its low immunogenicity dates back to 1981, when Akle et al. (1981) demonstrated the lack of immunogenicity of human amniotic epithelial cells: in fact, these cells do not express HLA-A, B, C, DR antigens or β_2_-microglobulin on their surface. The basement membrane is involved in the migration of epithelial cells (Terranova and Lyall 1986) and promotes their differentiation (Guo and Grinnell 1989) and uniform stratification. AM was also suggested as the ideal biological substrate for limbal epithelial stem cells (Grueterich et al. 2003); more specifically, AM prevents apoptosis, promotes adhesion of various cells and helps maintain normal epithelial cell morphology (Kurpakus et al. 1992; Ohno-Matsui et al. 2005). AM can be used as a temporary graft and functions as a basement membrane substitute in the eye; in fact, the presence of several growth factors in the AM tissue, together with its function as a structural support providing a basal membrane for new cell growth, are believed to be the main mechanisms by which the procedure achieves its therapeutic effects. Another important property of AM is its ability to inhibit vascularization. Hao et al. (2000) identified several anti-angiogenic and anti-inflammatory proteins in the AM. It is of note that the cornea and vitreous are avascular tissues, which is why this characteristic effect of AM is advantageous. Besides the beneficial physical properties of AM, the possibility of freezing and preserving AM patches has further expanded their clinical utilization. AM treatment is effective not only due to its clinical and pathological properties, but also its availability and ease of collection and preservation. Storage for months in tissue banks offers sufficient time to plan and perform surgeries or clinical trials (Dua and Azuara-Blanco 1999). Long term and short term storage methods were previously reported, including freeze-drying, storage in media up to 28 days (Hennerbichler et al. 2007). It was shown that short term preservation can better conserve the cells viability. At our tissue bank, AM patches are stored and preserved at temperatures of below −140 °C for up to 5 years. Many reports in the literature described the storage of AM at −80 °C and, depending on usage rates, AM has been used up to 6 months (Madhavan et al. 2002) and even 1 year (Dekaris and Gabrić 2009) after preparation. Additionally, storage for an indefinite period has also been suggested.

In this report, we describe pathological conditions treated with human AM showing statistical data from long term treatment. Transplantation of AM in an eye with a chronic neurotrophic corneal ulceration is presented as an example of the most frequent application of AM in ophthalmology; corneal ulcers. To manage the safety issues common to all transplantation procedures and provide a safe, sterile and consistent product, the methods used for screening, procurement, manipulation and banking AM tissue must be carefully set up, standardized and validated. The Tissue Bank Foundation of Treviso, in association with the Veneto Eye Bank Foundation, has been working on these methods for several years, refining and optimizing operations. We now present our experience in recovering and cryopreserving membrane patches in compliance with strict specifications designed to raise the standards and quality of the membrane patches.

## Materials and methods

### Donor selection and screening

Donors were selected on the basis of strict criteria including guidelines for harvesting, processing and distributing tissues for transplantation as approved by the National Transplant Centre. Selection criteria include absence of history of malignancies, absence of malformations or pathological conditions of the baby, gestation period of at least 35 weeks, negative medical history of the family for genetic diseases, and lifestyles of both parents not at risk for infectious diseases. The donor is also screened during pregnancy for neurodegenerative diseases and acute infections. The blood of the donors was also screened for HIV-1 and -2 antibodies, HTLV-1 and -2 antibodies, Hepatitis B Surface Antigen and Hepatitis B Core Antibody, Hepatitis C virus (HCV) and syphilis. Screening also included IgM/IgG antibodies against toxoplasmosis and cytomegalovirus and nucleic acid amplification tests (NAT) for HIV, HBV and HCV. A sample of the donor’s serum was frozen and stored for further investigation as required.

### Isolation, preparation and cryopreservation of amniotic membrane patches

The placenta was sourced from donors undergoing caesarean sections and processed shortly after retrieval. The AM was carefully detached from the chorion and rinsed with sterile saline solution to remove residual blood. The membrane was flattened on a nitrocellulose membrane filter (Merck Millipore), with its stromal/mesenchymal side facing down, in contact with the filter. The AM was immersed in a cocktail of antibiotics: vancomycin 50 µg/ml (Hospira), polimyxin B 100 µg/ml (Biochrom), ceftazidime 240 µg/ml (Fresenius-Kabi), lincomycin 120 µg/ml at +4 °C for 24 h in sterile conditions. Antibiotics are dissolved in RPMI 1640 Medium without phenol-red (Gibco, Life technologies). The AM was then cut into pieces 3 × 3 cm^2^ and immersed in 4 ml RPMI-1640 medium without phenol-red (Gibco, Life technologies), supplemented with 10 % human serum albumin and 10 % dimethyl sulfoxyde (DMSO, Cryo Sure, WAK-Chemie Medical GmbH) as cryopreservant. The patches were rolled up and inserted into cryovials, which were then filled with the cryopreservation medium/DMSO. Cryopreservation was achieved using a programmable cryogenic freezer (Planer KryoSave Integra, 750-30), which triggers a controlled cooling rate, taking about an hour an half to lower the temperature from +4 °C to −140 °C. The AM patches were stored in vapor-phase liquid nitrogen at −180 °C.

### Thawing and transportation

When required, cryovials containing the AM patches were thawed in a water bath at 60 °C, the nitrocellulose filter papers were changed, and the AM patch was repeatedly rinsed with sterile saline solution to wash away the cryopreservation media. The membrane patch was placed in a sealed container with 100 ml of transport medium (RPMI 1640 without phenol-red), which can be kept at 4 °C for up to 72 h before surgery. No antibiotics are added to the transport media to avoid any adverse effect on the eye.

### Microbiological analysis

In order to minimize the risk of contamination, microbiological analyses were performed at several stages throughout the process. Initial/primary placenta, transport medium, rinsing medium, membrane at the end of the process, medium at the end of the process, cryopreservation medium, rinsing medium after thawing, and transport medium from the tissue bank to the surgery room, were all tested.

### Distribution and patients

During the period 2003–2014, a total of 5604 membrane patches were collected from 215 placentas; of these, 5349 membranes were distributed to 220 centers throughout Italy. Patients were enrolled after being extensively informed about the treatment and giving their written consent to the procedure, in compliance with the Italian law. Various pathological conditions were reported in terms of the class and severity of the ocular defect. AM patches were supplied to all the doctors who requested them and were provided together with donor specifications and a questionnaire for reporting adverse events.

### Surgery and follow-up

Depending on the individual patient’s medical condition, surgeries included preliminary steps such as cleansing, rinsing or removal of excessive tissue, along with the preparation of the eye for AM transplantation. Surgeries were performed under local or peribulbar anesthesia. The AM was cut to the required size and sutured to the ocular surface with the epithelial surface facing upwards and the mesenchymal side in direct contact with the eye to ensure secure adhesion. For corneal ulceration the membrane was positioned over the defect and sutured to the surrounding cornea with 10/0 nylon sutures. This method allows the membrane to cover the surface with epithelial cells, which are then incorporated into the corneal tissue (Lee and Tseng 1997). For deep ulcers, a multilayer AM transplantation technique was adopted (Kruse et al. 1999). In patients with deep ulcers and herpetic keratitis (HK), epithelial debridement was performed and loosely adherent epithelium was removed before surgery. In band keratopathy a superficial calcium deposit was removed before rinsing with 4 % EDTA solution. In patients with subtotal stem cell deficiency associated with conjunctivalization, a 2 mm retrocession was performed and the fibrovascular pannus was removed by superficial dissection. After positioning to cover the entire corneal surface, the AM was sutured to the episclera. Unlike the technique used for treating corneal ulceration, in this procedure the epithelium was allowed to grow underneath the membrane. Conjunctival flaps were placed above the membrane to avoid penetration by fibrovascular tissue and to promote re-epithelialization. A temporary tarsorrhaphy protected the AM in these patients (Rama et al. 2001).

Post-surgery, patients were given 0.5 % cortisol for 1 week, together with fluoroquinolone (0.2 % ciprofloxacin) four times a day. The regimen was then reduced to decreasing cortisol doses for the following 3 weeks. Patient follow-up examinations were performed 1, 3, 6 and 12 months after surgery. This included an assessment of symptoms such as pain, photophobia and inflammation. Intraocular pressure and visual acuity were also measured, and re-epithelialization was assessed by fluorescein staining. Standard photography and slit-lamp biomicroscopy were used to evaluate corneal opacification and thickness.

## Results

### Distribution and pathological conditions treated

During the 12-year follow-up, 5604 amniotic membranes were isolated from 215 placentas, of which 5192 membrane patches were used for transplantation, 342 were discarded and 70 were selected for research. The recovery and banking procedure was successful in almost all cases except when the patch was physically damaged due to contact with vials during cryopreservation. None of the distributed membranes were positive for contamination after thawing, demonstrating the reliability of the microbiological controls performed during tissue collection. Table 1 shows that 183 membrane patches were discarded due to poor physical, histological or morphological conditions before preservation, 159 membrane patches were discarded after delivery to hospital due to damage or specific surgical reasons, and 70 membrane patches were selected for research purposes. The data also show that the majority of tissues collected were used for medical or surgical procedures (92.65 %). As indicated in Table 1, out of the total number of AM collected in the period, only 6.10 % were discarded due to physical damage after preservation, low histological quality or by the surgeon, while 1.25 % were used for research purposes. Table 2 includes a list of pathological conditions for which surgeons used AM each year over a period of 12 years between 2003 and September 2014. During this time a total 5349 surgical procedures were successfully performed using membrane patches distributed by the tissue bank. Corneal ulceration accounted for almost half of the cases (45.43 %). Other common conditions treated with AM were keratitis, endophthalmitis, chemical trauma and neoplasm, among others. Details on the use of AM for different pathological conditions are reported in Table 2.

**Table 1:** AM utilization percentages

Utilization	2003	2004	2005	2006	2007	2008	2009	2010	2011	2012	2013	2014^a^	Total
Grafted	345	384	426	453	437	467	489	533	475	458	429	296	5192
Sent then discarded	20	15	9	11	3	6	12	4	30	16	23	10	159
Discarded	11	18	9	4	7	17	5	11	12	9	15	65	183
Research	8	12	20	7	7				6	10			70
Total	384	429	464	475	454	490	506	548	523	493	467	371	5604

Number of AM used for transplantation, discarded or used for research between 2003 and 2014 (^a^ up to 30/09/2014)

**Table 2:** Distribution and pathological conditions treated during the period 2003–2014

Pathological conditions	2003	2004	2005	2006	2007	2008	2009	2010	2011	2012	2013	2014^a^	Total
Corneal ulcers including neurotrophic keratitis	142	160	182	199	179	219	256	235	240	244	214	160	2430
Keratitis/endophthalmitis	38	40	27	29	36	29	29	33	53	19	14	16	363
Pterygium	14	23	34	41	30	38	39	52	16	22	26	8	343
Post keratoplasty, glaucoma or cataract	21	22	28	38	32	28	28	50	36	30	19	7	339
Chemical trauma	32	32	41	41	31	35	30	18	32	22	11	7	332
Bullous keratopathy	17	27	16	21	36	32	31	26	25	14	11	9	265
Neoplasm of the ocular surface	12	20	22	20	15	11	21	28	18	23	26	23	239
Reconstruction of the conjunctiva and fornix	15	10	14	19	8	8	10	13	10	15	24	8	154
Corneal degeneration	5	7	11	4	3	11	8	12	15	11	21	15	123
Recurrent epithelial erosion	12	6	10	8	6	7	10	9	6	7	18	10	109
Primary and secondary limbal stem cell deficiency	9	10	12	6	3		4		5	13	11	5	78
Mucous membrane pemphigoid	9	5	4	4	3	6	6	5	4	7	4	2	59
Dystrophy	5	8	3	2	5	9	2	10	4	2	1	7	58
Mechanical trauma	1	3		1	6	3	5	3	1		2		25
Stevens–Johnson or Lyell’s syndrome	3	1	1					1	4	7	3		20
Reconstruction of the anophthalmic cavity	1		2	1	3			3	2	3	1	1	17
Physical trauma			3	3				2	1	5	1	1	16
Eyelid reconstruction		2		1		1		1			3	3	11
Dysfunctional tear syndrome			1			1		3	1		1	2	9
Other	29	23	24	26	44	35	22	33	30	30	41	22	359

Data on the number of AM utilized for ocular conditions between 2003 and 2014 (^a^up to 30/09/2014)

### Postoperative follow-up

The clinical outcome for all cases was established with 1 year of postoperative follow-up. In general, success was determined based on the scope of surgery and the presence of one or a combination of the following criteria: resolution of inflammation, relief of symptoms, restoration of regular and stable corneal epithelium, and restoration of the structural integrity of the eye.

Partial success was defined as attainment of only two of the above criteria. Failure was defined as the absence of all of the above criteria. Table 3 shows the percentage of AM transplantation success, partial success and failure.

**Table 3:** Success rates for AM therapy

Pathological condition	Success (%)	Partial success (%)	Failure (%)
Corneal ulcers including neurotrophic keratitis	100		
Keratitis/endophthalmitis	90	10	
Pterygium	71	21	8
Post keratoplasty, glaucoma or cataract	100		
Chemical trauma	15	61	24
Bullous keratopathy	100		
Neoplasia of the ocular surface	50	17	33
Reconstruction of the conjunctiva and fornix	30	23	47
Corneal degeneration	100		
Recurrent epithelial erosions	85	10	5
Primary and secondary limbal stem cell deficiency	18	50	32
Mucous membrane pemphigoid	10	11	79
Dystrophy	100		
Mechanical trauma	100		
Stevens–Johnson or Lyell’s syndrome	19	19	62
Reconstruction of the anophthalmic cavity	100		
Physical trauma	70		30
Eyelid reconstruction	100		
Dysfunctional tear syndrome	100		
Other	29	17	54

Data on success rates for AM ocular therapies between 2003 and 2014

Therapeutic effects after treatment were variable and related to the type of pathological condition treated. The best results were obtained when the membrane was used to control inflammation and pain. In general, a higher success rate was attained when membrane transplantation was performed for its key therapeutic indication, i.e. persistent epithelial defect with stromal ulceration in patients with functional limbal stem cell deficiency. In such cases the procedure was able to promote re-epithelialization in the majority of the patients and the restoration of a stable corneal epithelium in ulcers caused by different pathological conditions. In some patients, the AM was successfully incorporated into the corneal tissue, as demonstrated by the persistence of membrane fragments that remained visible for several days after surgery. In these cases, the membrane displayed its ‘scaffold-like’ properties by providing a healthy and intact basal membrane on which the patient’s cells were able to proliferate and restore the original structure of the epithelium.

### Amniotic membrane transplantation in ocular ulcers

**Fig. 1:**
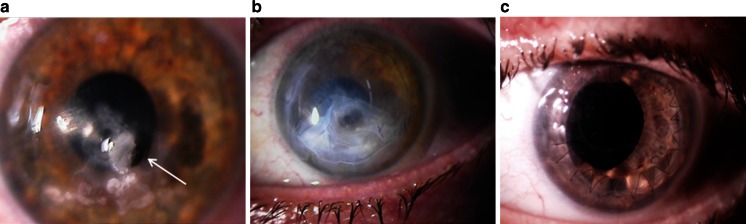
Treatment of corneal ulceration using AM. A patient with descemetocele *white arrow* caused by recurring HK (**a**). AM transplantation was performed and the gap was filled with membrane and glue (**b**). Images taken few weeks after filling and grafting. After AM grafting, penetrating keratoplasty was performed (**c**)

This report describes a successful AM transplantation in a patient with frequently recurring HK over a period of 3 years. The patient presented with a deep corneal perforation caused by neurotrophic ulcers leading to descemetocele formation (Fig. 1a). An inlay AM graft was performed, as well as an outlay AM graft (Fig. 1b). The gap was partially filled with cyanoacrylate glue, which also served to keep the graft in position. The graft assisted in preparing the patient for PK, which was successfully performed (Fig. 1c).

### Safety evaluation

No adverse reactions or other unexpected events related to AM were reported by the surgeons. To date, after a 12-year follow-up, the clinical history of AM distributed for transplantation does not indicate any harmful effects caused by AM, further confirming the high safety margin assured by this therapy. Quality controls include strict selection, analysis, processing and distribution procedures to guarantee the safety of the patches.

## Discussion

The purpose of the present study is to evaluate the role of human AM in the treatment of various conditions, in particular epithelial ulceration, pterygium, keratitis, glaucoma, bullous keratopathy (see Table 2). The benefits and safety features of AM in ocular disorders are evident, based on long term data analysis of these procedures. Also, as for the 12-year data and follow-up, our selection, isolation, pathological analysis, distribution and post-operative protocols were found to adhere to high standards, as reflected by the success rates for various procedures and surgeries. In the past, conjunctival autografts have been used with good clinical outcomes, but this procedure cannot be used in ocular cicatricial pemphigoid or Stevens–Johnson syndrome (bilateral diseases). The ocular surface is an extremely sensitive structure, whose health is crucial for the optimal functioning of the eye. The use of AM appears to be a useful alternative in the treatment of ocular surface disorders. AM has many important properties, including as an anti-fibrotic and anti-inflammatory agent. Fetal hyaluronic acid has been reported to suppress TGFβ signaling, which in turn inhibits the proliferation of conjunctival, corneal and limbal fibroblasts (Hao et al. 2000; Lee et al. 2000). In addition, AM has anti-angiogenic properties deriving from the production of several potent anti-angiogenic chemicals including thrombospondin-1, endostatin and all four tissue metalloproteinase inhibitors (Hao et al. 2000). The anti-microbial properties of AM have also been reported, with evidence of activity against both gram negative bacilli (Kjaergaard et al. 2001) and gram positive cocci (Kjaergaard et al. 1999). Beside its use in ophthalmology, AM has many applications in a range of medical conditions and procedures, such as diabetic foot ulcer grafts (Zelen et al. 2013), chronic burn wound dressings (Mohammadi et al. 2013), vaginoplasty (Fotopoulou et al. 2010) and myelomeningocele repair (Hasegawa et al. 2004). The ability of AM to promote healing of the epithelium may derive from the basement membrane’s tendency to facilitate the migration of epithelial cells, promote epithelial differentiation and reduce inflammation, scarring and vascularization. Based on the large number of cases reported here, the safety and effectiveness of AM transplantation for common ocular surface conditions can be demonstrated. AM may also represent a viable therapeutic alternative when conservative medical treatment fails. In particular, corneal ulceration is the most frequent condition treated by AM transplantation (45.43 %), with a 100 % success rate in 2430 patients, further highlighting the important role of AM in the treatment of ocular diseases. Lee and Tseng (1997) performed AM transplantation in 11 eyes of 11 consecutive patients with corneal ulcers; the corneal epithelial defects had persisted for a mean of 17.5 ± 13.9 weeks. Another report on the clinical use of AM in the treatment of corneal ulcers is by Kruse et al. (1999), who reported on the use of multiple layers of AM to reconstruct deep corneal ulcers in 11 patients; after a 1-year follow-up the defects had healed in 9 out of 11 patients (Kruse et al. 1999). In the case of keratitis (6.79 % of total cases), a success rate of 90 % was recorded; for pterygium (6.41 %), the success rate was 71 %; and for the treatment of surgical outcomes (6.34 %), the success rate was 100 %. The average success rate for all conditions was 69.25 %, however, for 10 conditions treated with AM the success rate was more than 90 %. This excellent feedback data can be credited to the properties of AM and probably also to the presence of a fraction of viable cells in cryopreserved AM preparations (Hennerbichler et al. 2007). However, it seems that the integrity of the matrix is more significant than the viability of the epithelium with regard to the biological properties of the membrane. Several AM denudation methods failed to prevent the growth of limbal stem cells, as recently reported (Zhang et al. 2013). Unlike other procedures by tissue banks (Dua and Azuara-Blanco 1999), our procedure contains a washing step prior to use of the AM. Although this can wash away many biologically active molecules, it is believed to be an important step for washing away DMSO and preservation media. AM can be preserved for a longer time and retains its activity when stored at 4 °C for up to 72 h, allowing for correct membrane preparation and transportation to institutions or hospitals, and providing sufficient time for preoperative planning. The success of the re-epithelialization process depends on the release of plentiful growth factors by a healthy population of AM cells. Less satisfactory results can be seen when AM transplantation is used to treat patients with severe limbal stem cell deficiency. In such cases the procedure is unsuccessful in promoting the regeneration of the epithelial layer, probably due to extensive damage to stem cells in the limbal region and their inability to repeatedly proliferate. For these patients, the treatment did provide some degree of relief from pain and inflammation, which was also useful as a preliminary step prior to limbal transplantation surgery.
